# Transplantation of older DCD livers in the machine perfusion era: a U.S. cohort study

**DOI:** 10.3389/ti.2026.16689

**Published:** 2026-05-29

**Authors:** Emmanouil Giorgakis, Ioannis A. Ziogas, Dimitrios Moris, Dimitrios N. Varvoglis, Charalampos Theocharopoulos, Dor Yoeli, Megan A. Adams, Andrew S. Barbas, Martin I. Montenovo, Melissa Chen, Sorabh Kapoor, Esteban Calderon, Andrew M. Moon, Sasha Deutch-Link, Hersh Shroff, Neil D. Shah, Oren K. Fix, A. Sidney Barritt, Amit K. Mathur, Trevor Nydam, Chirag S. Desai, Paulo N. Martins, Andrea Schlegel

**Affiliations:** 1 Division of Abdominal Transplantation, Department of Surgery, University of North Carolina at Chapel Hill School of Medicine, Chapel Hill, NC, United States; 2 Division of Transplant Surgery, Department of Surgery, University of Colorado Anschutz Medical Campus, Children’s Hospital Colorado, Aurora, CO, United States; 3 Department of Surgery, MedStar Georgetown Transplant Institute, Washington, DC, United States; 4 Division of Abdominal Transplant Surgery, Department of Surgery, Duke University Medical Center, Durham, NC, United States; 5 Division of Hepatobiliary Surgery and Liver Transplantation, Department of Surgery, Vanderbilt University Medical Center, Nashville, TN, United States; 6 Division of Gastroenterology and Hepatology, University of North Carolina at Chapel Hill School of Medicine, Chapel Hill, NC, United States; 7 Division of Transplant Surgery, Mayo Clinic, Phoenix, Arizona, NC, United States; 8 Transplant Institute, Department of Surgery, Division of Transplantation, University of Oklahoma, Oklahoma City, OK, United States; 9 Transplant Center, Digestive Disease and Surgery Institute, Cleveland Clinic Foundation, Cleveland, OH, United States

**Keywords:** donation after circulatory death, dynamic preservation, hypothermic oxygenated perfusion, liver transplantation, machine perfusion

## Abstract

Donation after circulatory death (DCD) livers increasingly use machine perfusion (MP). This study evaluates MP’s impact on older DCD livers based on data from the United Network for Data Sharing, covering all first adult DCD liver transplants (2016–2025). The cohort, divided into pre-MP and MP eras (separated by the FDA approval of the first normothermic MP platform in 2021), showed accelerated growth in DCD liver transplants during the MP era. Donors ≥60 rose 7.8-fold, including donors ≥70 (a USA first). By 2025, DCD livers accounted for 43.2%, with 61.35% from donors ≥50. Normothermic regional perfusion (NRP) (3.0%–21.7%), NMP (2.3%–35.6%), and sequential NRP-NMP (0.1%–19.2%) increased significantly (p < 0.001). The MP era was associated with a decrease in median waitlist time from 112 to 62 days (p < 0.001). Early graft survival was similar across ages. For ages 50–59, 1- and 3-year survivals were 87.9%/78.4% pre-MP and 90.1%/78.8% in the MP era. For 60–69, survival was 85.0%/80.6% pre-MP and 90.0%/71.3% in the MP era. DCD LTs for ≥70 were limited to the MP era with 87.8% 1-year survival. Multivariable Cox regression showed that static cold storage (HR = 1.19), donor age 50–69 versus 18–49, and recipient age (HR = 1.04) increased the risk of graft loss after adjustment. MP is associated with an increased number of older DCD liver transplants and acceptable early graft survival.

## Graphical Abstract

**Figure:**
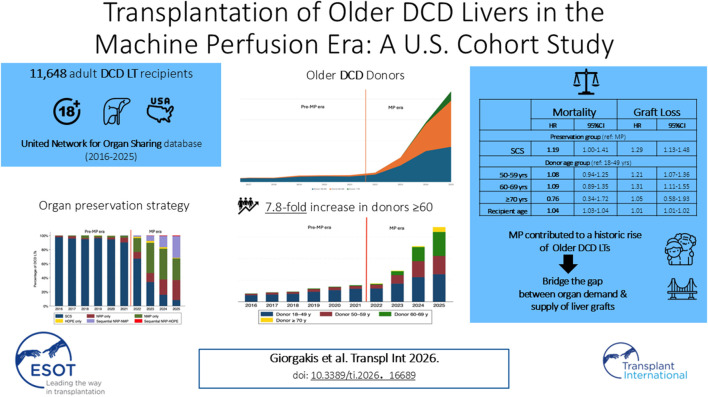

## Introduction

Donor age is a recognized risk factor in liver transplantation (LT) [1, 2]. Early studies showed higher biliary complications in donors >40 years [3]. Later cohort studies found that DCD livers from donors aged >60 or 70 years had comparable outcomes when other risks were limited [4–7]. Studies from the United Kingdom suggested that donor age >60 alone did not predict posttransplant survival [7]. However, DCD outcomes declined when these donors had other risk factors, supporting the concept of a cumulative DCD donor risk profile [7–9]. In 2021, the International Liver Transplantation Society DCD LT Consensus Conference in Venice recommended the selective utilization of livers from donors >60 years with consideration of other risk factors, such as donor functional warm ischemia time (WIT), body mass index (BMI), macrovesicular steatosis, donor hepatectomy time, and projected cold ischemia time (CIT) [10].

Until very recently, in the USA, older DCD liver grafts were considered those from donors ≥50 years [4, 5]. Only a small proportion of transplanted DCD liver grafts originated from donors ≥60. Most transplant centers traditionally refrained from using older livers [6, 11, 12].

The global DCD donor landscape changed drastically with the broad adoption of dynamic preservation of these organs [13]. Randomized controlled trials (RCTs) and large-scale registry analyses have demonstrated that normothermic machine perfusion (NMP) enhances the safety of expanding the use of older DCD livers by improving graft viability, reducing post-transplant complications, and increasing organ utilization through better viability assessment [14, 15]. Normothermic regional perfusion (NRP) restores *in-situ* regional organ circulation after the DCD donor’s death, enabling the safer use of older grafts [16–22]. NRP is thought to achieve this by facilitating early tissue reoxygenation after declaration of death, thereby replenishing the organ’s energy stores before preservation. Other techniques include hypothermic oxygenated perfusion (HOPE), for which there is strong evidence of improved graft survival and fewer adverse events in extended-criteria donors [23]. In the USA, utilization of HOPE has largely been limited to clinical trials, as no HOPE devices were FDA-approved until January 2026 [24].

Using a large retrospective United Network for Organ Sharing (UNOS) cohort, we aimed to evaluate the effects of MP on older DCD liver utilization in the USA.

## Materials and methods

Data were obtained from the UNOS Standard Transplant Analysis and Research data file [25]. The UNOS database administers the Organ Procurement and Transplantation Network (OPTN) under a contract with the US Department of Health and Human Services. No Institutional Review Board approval was required as all data were publicly available in a de-identified form.

This retrospective cohort study included all DCD LTs performed in adult (≥18 years) recipients from adult donors in the USA between January 1st, 2016, and December 31st, 2025. Multi-organ transplants and re-transplants were excluded. The cohort was divided into two eras. September 28th, 2021, was used to define the pre-MP and MP eras, based on the Food and Drug Administration (FDA) approval of the first commercially available NMP platform in the USA. Prior to this date, MP cases in the USA were limited to centers participating in related trials. All HOPE cases included have been in the context of a clinical trial. The first USA reports of NRP LTs were published in 2022 [26, 27]. Cohorts were further subdivided into donor age groups: 18–49, 50–59, 60–69, and ≥70 years. MP modality groups were defined as: static cold storage (SCS), NRP, NMP, HOPE, sequential NRP-NMP, and sequential NRP-HOPE.

WIT was defined as the interval from withdrawal to cross-clamping. CIT was defined as the period during which the organ was preserved on ice. Total preservation time (TPT) was defined as the time from cross-clamp to organ reperfusion; therefore, TPT encompasses SCS (CIT) and MP times. As in prior studies, donors were considered to have undergone NRP if WIT was ≥40 min [27].

### Statistical analysis

Categorical variables were reported using frequencies and percentages. Continuous variables were reported using medians and interquartile ranges. Group differences were assessed using the Chi-square test for categorical variables and the Mann-Whitney U test for continuous variables. Pearson’s correlation coefficient was used to evaluate changes in the percentage of each preservation group and donor age group over time.

Post-LT patient and graft survival were the primary outcomes. Patients were censored at the last follow-up. The Kaplan-Meier method was used to estimate patient and graft survival. Log-rank test was used to assess differences in post-transplant survival across graft types. Further analysis using multivariable Cox regression evaluated survival by preservation group (for parsimony, defined as SCS vs. non-SCS), adjusting for confounding factors. The variables included in the multivariable Cox regression were selected *a priori* based on biological importance and data availability to avoid the inferential limitations of selecting variables for multivariable models based on univariable comparisons or stepwise procedures [28]; these were preservation group, WIT, donor age group, donor and recipient BMI, recipient age, laboratory Model for End-stage Liver Disease (MELD) score, and recipient transplant indication/diagnosis. Cohort development and statistical analyses were conducted using Stata IC 19.0 (Stata Corp LLC, College Station, Texas, US).

## Results

### Demographics

**FIGURE 1:**
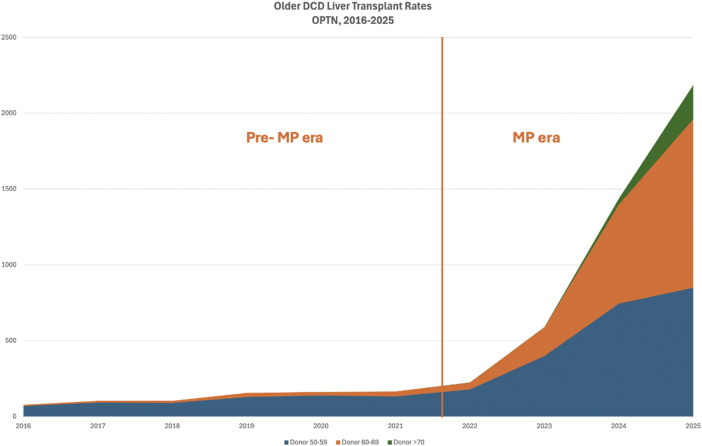
Temporal trends in DCD liver transplantation from older donors (2016–2025, OPTN data). The vertical line marks the transition from the pre-machine perfusion (MP) to the MP era.

**FIGURE 2:**
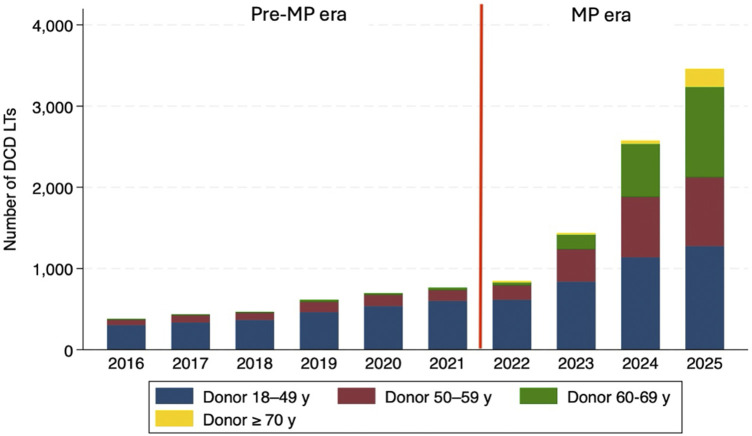
Temporal trends in DCD liver transplantation categorized by donor age group (2016–2025, OPTN data). The vertical line marks the transition from the pre-machine perfusion (MP) to the MP era.

N = 11,648 DCD LTs. Although the annual use of DCD livers, particularly older DCD liver grafts, increased across both eras, this augmentation was exponential in the MP era (Figures 1, 2).

In the early period, 77.2% of DCD livers were from donors aged <50 years, with the remainder aged 50–69. No DCD livers from donors aged ≥70 were used before 2021 (Table 1).

**TABLE 1:** Demographic and clinical characteristics by era.

Variable	Pre-MP era (n = 3,308) Median (IQR)	MP era (n = 8,340) Median (IQR)	Total (n = 11,648) Median (IQR)	p-value
Recipient age (years)	59.0 (52.0–65.0)	59.0 (50.0–65.0)	59.0 (51.0–65.0)	0.07
Waitlist time (days)	112.0 (28.0–271.0)	62.0 (16.0–206.0)	72.0 (18.0–222.0)	<0.001
Recipient BMI (kg/m^2^) (n = 11,646)	28.7 (25.2–33.1)	28.7 (25.0–33.0)	28.7 (25.0–33.0)	0.73
Diagnosis	​	​	​	<0.001
HCC	901 (27.2%)	1,720 (20.6%)	2,621 (22.5%)	​
MASLD	628 (19.0%)	1,726 (20.7%)	2,354 (20.2%)	​
Alcohol-associated liver disease	936 (28.3%)	2,996 (35.9%)	3,932 (33.8%)	​
Other	843 (25.5%)	1,898 (22.8%)	2,741 (23.5%)	​
Laboratory MELD score (n = 11,645)	18.0 (13.0–24.0)	19.0 (14.0–25.0)	19.0 (14.0–24.0)	<0.001
Donor age group	​	​	​	<0.001
18–49	2,554 (77.2%)	3,889 (46.6%)	6,443 (55.3%)	​
50–59	639 (19.3%)	2,178 (26.1%)	2,817 (24.2%)	​
60–69	115 (3.5%)	2,007 (24.1%)	2,122 (18.2%)	​
≥70	0 (0.0%)	266 (3.2%)	266 (2.3%)	​
Donor BMI (kg/m^2^) (n = 11,620)	26.7 (23.6–31.1)	28.2 (24.1–33.0)	27.7 (24.0–32.4)	<0.001
Preservation group	​	​	​	<0.001
SCS	3,127 (94.5%)	1,770 (21.2%)	4,897 (42.0%)	​
NRP	101 (3.0%)	1,811 (21.7%)	1,912 (16.4%)	​
NMP	75 (2.3%)	2,972 (35.6%)	3,047 (26.2%)	​
HOPE	2 (0.1%)	130 (1.6%)	132 (1.1%)	​
Sequential NRP-NMP	3 (0.1%)	1,605 (19.2%)	1,608 (13.8%)	​
Sequential NRP-HOPE	0 (0.0%)	52 (0.6%)	52 (0.5%)-	​
Total warm ischemia time (minutes)	23.0 (19.0–27.0)	31.0 (24.0–99.0)	27.0 (22.0–74.0)	<0.001
Total preservation time (hours) (n = 11,326)	5.3 (4.4–6.3)	14.5 (6.8–19.1)	8.9 (5.2–17.2)	<0.001

BMI, body mass index; HCC, hepatocellular carcinoma; HOPE, hypothermic oxygenated perfusion; IQR, interquartile range; MASLD, metabolic dysfunction-associated steatotic liver disease; MELD, model for end-stage liver disease; MP, machine perfusion; NMP, normothermic MP; NRP, normothermic regional perfusion; SCS, static cold storage.

In the late period, annual DCD LT volume growth accelerated (Figure 2), with significant increases in donors aged 50–59 (from 19.3% to 26.1%) and 60–69 (from 3.5% to 24.1%). Donors ≥60 increased from 3.5% to 27.3% – a 7.8-fold rise–, including 266 (3.2%) DCD donors aged ≥70; a USA first. The proportion of younger donors (18–49 years) declined from 77.2% to 55.3%.

**FIGURE 3:**
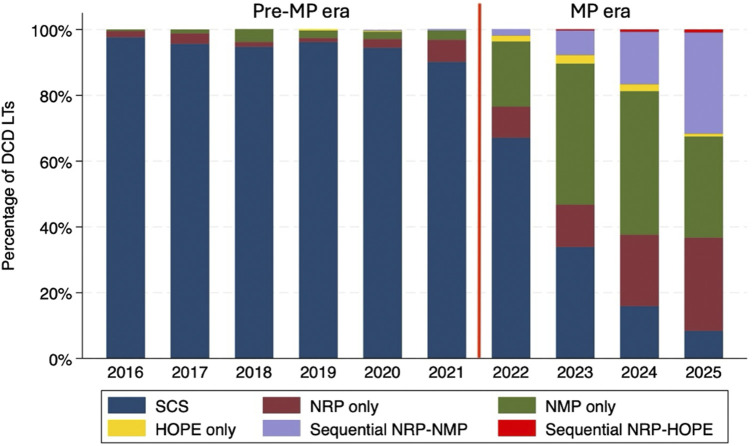
Bar plot demonstrating the percentages of each machine perfusion (MP) modality amongst all DCD LTs.

During our study period, total LT rates increased by 62.7% (from 8,497 LTs in 2016 to 13,824 in 2025). By the end of 2025, DCD allografts (n = 5,669) accounted for 43.2% of deceased-donor allografts (n = 13,116). In the MP era, donors ≥50 (n = 4,451) accounted for the largest share of DCD donors (61.35%) (Figure 2). DCD SCS dropped from 94.5% to 21.2% in the MP era, while there was a steep rise in NRP (3.0% vs. 21.7%), NMP (2.3% vs. 35.6%), and sequential NRP-NMP deployment (0.1% vs. 19.2%) (p < 0.001) (Figure 3, Table 1).

The MP-era was associated with 44.6% decrease in median waitlist time (112–62 days, p < 0.001) (Table 1). The primary LT indication shifted from hepatocellular carcinoma (27.2%) to alcohol-associated liver disease (35.9%) (p < 0.001). The median donor WIT increased from 23.0 to 31.0 min.

### Survival outcomes

**FIGURE 4:**
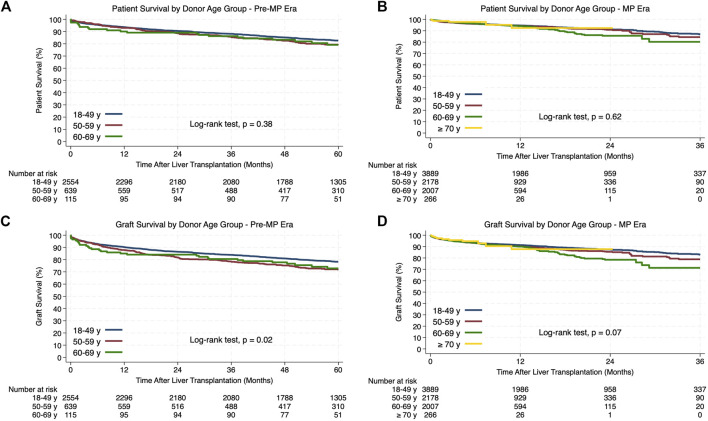
Kaplan-Meier patient and graft survival curves by donor age group in the pre-machine perfusion (MP), **(A,B)**, and MP eras **(C,D)**.

In the pre-MP era, graft survival was higher in donors aged 18–49 (Figure 4C). Early graft survival was comparable across all age groups in the MP era (Figure 4D). No statistically significant difference in patient survival was observed amongst the three donor age groups (Figures 4A,B).

For the 50–59-year cohort, 1- and 3-year graft survival was 87.9% and 78.4% in the pre-MP era (n = 639), and 90.1% and 78.8% in the MP era (n = 2,178), respectively (Table 2). For the 60–69-year group, 1- and 3-year graft survival was 85.0% and 80.6% in the pre-MP era (n = 115), and 90.0% and 71.3% in the MP era (n = 2,007), respectively (Table 2). Utilization of ≥70-year-old DCD livers was reported only in the MP era, with 1-year survival of 87.8% (Table 2).

**TABLE 2:** Benchmark point estimates of unadjusted cumulative patient and graft survival after liver transplantation.

​	Donor 18–49 years	Donor 50–59 years	Donor 60–69 years	Donor ≥70 years
Pre-MP era	(n = 2,554) % (SE)	(n = 639) % (SE)	(n = 115) % (SE)	(n = 0) % (SE)
Patient Survival				
1 year	93.6% (0.5)	93.0% (1.0)	90.1% (2.8)	-
3 years	88.2% (0.7)	85.5% (1.4)	86.3% (4.0)	-
5 years	82.7% (0.8)	79.1% (1.7)	79.1% (4.2)	-
Graft survival				
1 year	90.2% (0.6)	87.9% (1.3)	85.0% (3.4)	-
3 years	93.9% (0.7)	78.4% (1.6)	80.6% (3.7)	-
5 years	78.3% (0.8)	71.9% (1.8)	72.9% (4.4)	-
Post-MP era	**(n = 3,889)**	**(n = 2,178)**	**(n = 2,007)**	**(n = 266)**
Patient survival				
1 year	94.6% (0.4)	94.2% (0.6)	94.1% (0.7)	92.4% (3.9)
3 years	86.8% (1.1)	84.4% (2.1)	80.2% (4.1)	-
Graft survival				
1 year	91.4% (0.5)	90.1% (0.8)	90.0% (0.9)	87.8% (4.3)
3 years	82.9% (1.1)	78.8% (2.1)	71.3% (4.4)	-

Table entries are estimates of cumulative patient and graft survival percentages (standard errors).

MP, machine perfusion; SE, standard error.

Bold values indicate the number (n) of transplanted patients in each donor category.

In multivariable Cox regression, SCS (hazard ratio [HR] = 1.19, 95% confidence interval [95% CI]: 1.00–1.41, p = 0.045) and increasing recipient age (HR = 1.04, 95% CI: 1.03–1.04, p < 0.001) were associated with increased risk of mortality when adjusted for donor age group, WIT, donor BMI, recipient BMI, diagnosis, and laboratory MELD score (Table 3). SCS (HR = 1.29, 95% CI: 1.13–1.48, p < 0.001), donor age 50–69 years (vs. 18–49 years), and increasing recipient age (HR = 1.01, 95%CI: 1.01–1.02, p < 0.001) were also associated with increased risk of graft loss when adjusted for WIT, donor BMI, recipient BMI, diagnosis, and laboratory MELD score (Table 3).

**TABLE 3:** Multivariable Cox regression models for patient mortality and graft loss.

Variable	Mortality			Graft loss		
	Hazard ratio	95% CI	p-value	Hazard ratio	95% CI	p-value
Preservation group (ref: non-SCS)	-	-	-	-	-	-
SCS	1.19	1.00–1.41	0.045	1.29	1.13–1.48	<0.001
WIT (min)	1.00	0.99–1.00	0.33	0.99	0.99–1.00	0.87
Donor age group (ref: 18–49 years)	-	-	-	-	-	-
50–59 years	1.08	0.94–1.25	0.29	1.21	1.07–1.36	0.002
60–69 years	1.09	0.89–1.35	0.39	1.31	1.11–1.55	0.001
≥70 years	0.76	0.34–1.72	0.51	1.05	0.58–1.93	0.86
Donor BMI (kg/m^2^)	1.00	0.99–1.01	0.93	1.00	0.99–1.01	0.88
Recipient age (yrs)	1.04	1.03–1.04	<0.001	1.01	1.01–1.02	<0.001
Recipient BMI (kg/m^2^)	1.00	0.99–1.02	0.41	1.01	0.99–1.01	0.19
Laboratory MELD	1.01	0.99–1.02	0.06	1.01	0.99–1.01	0.11
Diagnosis (ref: malignancy)	-	-	-	-	-	-
MASLD	0.98	0.83–1.17	0.83	1.02	0.88–1.18	0.80
Alcohol-associated liver disease	0.92	0.77–1.09	0.33	0.93	0.80–1.07	0.31
Other	0.90	0.76–1.07	0.26	0.92	0.79–1.07	0.30

BMI, body mass index; CI, confidence interval; MASLD, metabolic dysfunction-associated liver disease; MELD, model for end-stage liver disease; SCS, static cold storage; WIT, warm ischemia time.

## Discussion

DCD allograft use, although slowly rising in the 2010s, entered a rapid growth phase since 2021, evolving from a peripheral (7.4% in 2016) to a dominant source of liver supply. A staggering 5,669 (43.2%) of all deceased-donor recipients received a DCD liver graft in 2025. Older DCDs have evolved into the fastest-growing donor population (Figure 1) [29].

As the name implies, the MP era was defined by the rapid, broad-scale adoption of dynamic perfusion and preservation techniques of DCD livers, and, reversibly, a marked decline in SCS. The remarkable rise in overall LTs was driven largely by increased use of DCDs, particularly from older donors. The MP era also coincided with increased rates of LT for alcohol-associated liver disease, currently the main indication for LT in the USA (Table 1). This transition may have been catalyzed by the more liberal use of DCD livers, thereby freeing up DBD grafts for higher-MELD patients, and by growing confidence in the use of pumped DCD allografts in sicker patients.

With a turning point in 2021, older DCD donors have moved from a marginal donor group to the primary driver of DCD LT volumes. In the pre-MP era, approximately 80% of DCD livers were from donors <50 years, with the remainder aged 50–69; no transplanted livers were from donors ≥70. In the MP era, the share of DCD donors aged ≥50 increased more than 2-fold (from 19.3% to 53.4%). DCD donors ≥60 rose by 7.8-fold (from 3.5% to 27.3%) (Table 1). The utilization of older DCD donors (≥50 years) has outpaced that of younger DCD donors, becoming the dominant DCD donor source in 2025 (n = 3,478; 61.3%) (Figures 1, 2).

Early graft survival was comparable across DCD donor age groups in the MP era, unlike in the earlier period (Table 3; Figure 4). There was a drop in 3-year graft survival in the 60–69 years age group across the two eras (80.6% vs. 71.3%, respectively), attributable to a very selective use of these organs in the early period (stringent donor selection bias), and reversibly, the liberal use—approaching a twentyfold increase—of older DCDs in the current era. Although not explicitly linked to the advent of MP technologies—considering the presence of several other confounding developments in transplant care during the same period—the waitlist time for liver transplantation (LT) nearly halved during the MP era (p < 0.001, Table 1).

### Older DCD liver grafts in the pre-MP era

Before MP widespread adoption, the standard organ preservation approach was SCS, which involved flushing the organ with preservation solution at 4 °C and transporting it under cold conditions (Table 4). Older DCD liver grafts were associated with high rates of ischemic biliary complications, EAD, post-reperfusion syndrome, and graft loss [14, 30, 31]. Donors ≥40 years and extended CIT appeared to further increase this risk [3]. Post-reperfusion syndrome, more common in DCD LT recipients, was associated with higher rates of acute kidney injury and decreased 5-year graft survival [31].

**TABLE 4:** Comparison of machine preservation modalities for older DCD livers.

Method	Use in US	Key mechanisms	Impact on utilization	Clinical outcomes	Evidence in older DCD livers	Advantages	Disadvantages
SCS	Historically standard	Hypothermic metabolic arrest	High discard rates (>30% for older DCD)	High risk of EAD, NAS, PNF	Poor outcomes in older DCD (contrary to the UK where outcomes were comparable in donors 60 and 70 years after appropriate selection); limited tolerance for ischemia	Widely available, inexpensive, simple logistics	High discard, poor outcomes in older donors, no viability testing, high NAS risk; time- constrained
HOPE	One device FDA approved 1/2026; increasingly used in trials and select centers; portable device (e.g., PILOT trial)	Oxygenated hypothermic perfusion: preserves mitochondria, reduces ROS	Reduces discard rates to <10%	↓ NAS, ↓ EAD, improved survival; ↓ retransplantation	Strongest evidence for reducing biliary complications; 5-year graft survival ∼81%	Strong biliary protection, reduces discard, improves graft survival, feasible logistics; FMN viability testing, cost-effective (European data)	FDA-approved 1/2026; requires device/oxygen
NMP	FDA-approved; rapidly expanding	Maintains physiologic metabolism; it can be applied upfront vs. endischemic	Discard reduced (7.2% vs. 30.5% with SCS)	↓ EAD, ↓ PNF, improved hemodynamics, resource sparing, differs between Back-to-Base and upfront	Enables safe use of >60 years old donors; less biliary protection than HOPE	Allows viability testing, prolongs preservation, improves perioperative stability; permits more daytime transplants	Costly, less effective for biliary protection than HOPE, requires blood products/logistics
NRP	Limited adoption due to logistics and regulatory barriers	Restores *in situ* donor circulation before retrieval	Expands acceptance rates; sequential NRP on donors >60 years and longer warm ischemia time	↓ NAS, ↓ EAD, improved 1-year survival	Effective for older donors, strong biliary protection if WIT not too long	Protects graft *in situ*, reduces ischemia, strong outcomes in older donors	Logistically complex, ethical/regulatory hurdles, requires ECMO equipment; resource intensive; time-constrained
Sequential MP (NRP + *ex-situ* MP, HOPE-NMP, HOPE-COR-NMP)	Emerging, at high-volume centers	NRP followed by HOPE/ NMP	Further reduces discard; promotes use of >60 years donors	↓ NAS; improved 1-year survival	Promising for older DCDs; meta-analyses show superior outcomes vs. single modality	Combines benefits of NRP & HOPE/ NMP; best outcomes reported; enables viability testing and reconditioning	Limited availability, more complex logistics than HOPE alone, not widely used, costlier

COR, continuous oxygenated rewarming; DCD, donation after circulatory death; DHOPE, dual hypothermic oxygenated machine perfusion; ECMO, extracorporeal membrane oxygenation; FMN, flavin mononucleotide; HMP, hypothermic oxygenated machine perfusion; NAS, non-anastomotic strictures; NMP, normothermic machine perfusion; NRP, normothermic regional perfusion; SCS, static cold storage; PNF, primary nonfunction.

These early reports defined the DCD practice in the USA for the following decade (2010–2020). Transplant centers adopted strict donor selection criteria, often excluding liver grafts from donors >50^5^. Most of these organs were more likely to be discarded rather than transplanted [14, 19, 32]. During the same period, older DCD liver grafts were used in Europe, albeit selectively, with acceptable outcomes [7, 32, 33]. The UK has historically had the largest cohort of cold-stored DCD LTs without MP, with favorable outcomes [6, 34, 35].

### Older DCD liver grafts in the MP era

The use of older DCD donors reached a pivotal juncture in 2021, which coincided with the FDA approval of the TransMedics ® Organ Care System (OCS) platform (September 2021), followed by the FDA approval of OrganOx metra® System (December 2021), and the first publicized reports of NRP LTs in the USA (May 2022) [27, 29, 36]. By 2025, DCD livers accounted for 41.0% of LTs, a remarkable 5.5-fold increase over a decade (Figure 1). This sharp rise indicates a major shift in transplant practice and the acceptance of older DCD liver grafts in the USA. Similar trends have been observed globally, making older DCD donors the latest Frontier in the organ donor pool [13].

NMP enhances the use of older DCD liver grafts by enabling real-time viability assessments and reducing organ discard rates (7.25% vs. 30.52% for SCS) [37, 38]. NMP also reduces the incidence of EAD [31, 39–41]. However, according to the European experience, its effect on long-term graft survival and biliary complications is less pronounced than that of HOPE [42] (Table 4).

Following the first landmark USA NRP cohort publications [20, 22], NRP utilization increased from 3% to 21.7%, and to nearly 41.5% when including sequential NRP. NRP has been associated with significant reductions in NAS, PNF, and EAD, as well as improved 1-year graft survival [20, 22, 31, 42]. Although it is increasingly adopted as the preferred approach in DCD procurement across the organ procurement organizations (OPOs), its logistical complexity often limits its use [31, 42] (Table 4).

HOPE improves 1-year graft survival and reduces retransplantation rates [43] (Table 4). Although long-term survival data are still emerging, large real-world cohorts of HOPE-treated DCD liver grafts, including those from older donors, report a 5-year death-censored graft survival rate of 81% and low rates of graft loss due to PNF or NAC, regardless of donor age or risk profile [23].

The latest US-based RCT (PILOT trial) on HOPE used a portable HOPE device under FDA and institutional review board oversight [44]. The trial confirmed safety and demonstrated improved early clinical function with HOPE, including in patients with DCD livers from donors >65^44^. These findings are consistent with broader evidence that HOPE reduces EAD and biliary complications in DCD livers, including those from older donors, compared to SCS [12, 44, 45]. This technology has only recently received FDA approval for standard-of-care use in LT in the USA [24].

Sequential NRP, i.e., NRP followed by *ex-situ* NMP or HOPE, has shown a lower rate of NAS compared to SCS and single-modality perfusion, even in grafts from donors ≥60 years [19, 42] (Table 4). Meta-analyses confirm that HOPE-based strategies, including sequential protocols, are associated with improved 1-year graft survival and reduced graft loss, as well as a lower risk of biliary complications compared to single-modality NMP or SCS [46, 47].

In conclusion, the widespread adoption of MP has lifted prior reservations about the broader use of older DCD livers. Although not explicitly associated with the introduction of MP—given that multiple simultaneous changes occurred during this period—nearly 50% of current DCD donors are aged 50 or older, including those aged 70 or above. This demographic constitutes the fastest-growing donor group in the US [29].

### Logistical and economic considerations

NMP requires a perfusion system, blood products, and specialized personnel, making it most suitable for centers that can absorb the higher acquisition costs and accommodate complex logistics [40, 41, 48]. NMP may be initiated at the donor hospital or applied at the recipient center. The latter (“end-ischemic” or “back-to-base” NMP) is less effective in terms of its ischemic bilioprotective effect due to the inevitable intervening cold ischemic period during SCS [14, 41].

The TransMedics RCT, a multicenter study evaluating the TransMedics® OCS for NMP, reported that OCS enabled the safe utilization of higher-risk and marginal donor livers, including a significantly higher proportion of DCD grafts, compared to SCS [49]. OCS recipients experienced fewer cases of reperfusion syndrome, less blood loss and transfusions, shorter surgeries, and shorter ICU and hospital stays. However, OCS organ acquisition costs were substantially higher. The median OCS organ acquisition cost was $135,930, compared to $50,940 for SCS, representing a nearly threefold increase [49]. Main cost factors include service, disposable perfusion components, and logistics of remote perfusion initiation at the donor hospital. Notably, these costs were not offset by reductions in other perioperative expenses during the index hospitalization, as overall hospitalization costs remained higher for OCS cases ($256,810 vs. $209,144 for SCS) [49] (Table 5).

**TABLE 5:** Logistical and economic considerations of machine perfusion modalities in older DCD liver transplantation.

Modality	Economic impact	Logistical requirements	Feasibility/Barriers in U.S.
HOPE	Cost-effective vs. SCS; ↓ ICU stay and intervention costs; cost-effectiveness achieved with ≥25–30 cases/year [50] (NL)	Perfusion device, oxygen supply, trained staff; protocol relatively simple and center-based	Most feasible for U.S. centers; FDA-approved 1/2026 for routine clinical use in DCD livers with no duration or prior cold storage duration limit
NMP	Higher acquisition/preservation costs [49]; 90-day healthcare costs comparable to SCS due to ↓ early complications for back-to-base NMP [48] (US)	Perfusion system which may be portable (upfront MP) or stationary (endischemic MP); blood products, specialized personnel; allows prolonged preservation and real-time viability testing	FDA-approved but higher complexity and costs limit broad use
NRP	Cost-effective [51]; potential ↓ complications and enhanced utilization	Requires extracorporeal membrane oxygenation (ECMO) equipment, multidisciplinary team, OPO coordination	Logistical, ethical, and regulatory barriers; increasing adoption by the OPOs and the individual centers in US; more experience in Europe

DCD, donation after circulatory death; HOPE, hypothermic oxygenated perfusion; NMP, normothermic machine perfusion; NRP, normothermic regional perfusion; NL, data from the Netherlands; OPO, organ procurement organization.

The back-to-base NMP model, particularly with devices such as OrganOx metra®, although it increases organ acquisition and preservation costs, is associated with fewer perioperative complications and does not increase overall short-term healthcare costs in DCD LT [48] (Table 5).

MP applications also enabled LT to transition from emergency to semi-elective daytime surgery, alleviating strain on the operating apparatus and expediting avoidance of costlier, out-of-hours complex procedures [52].

NRP use has increased over the recent yrs, with more OPOs standardizing its use in DCD procurements. Implementation involves extracorporeal membrane oxygenation equipment, specialized training, and coordination with the OPOs and across procurement teams, making it resource-intensive. However, it is cost-effective because it increases organ yield and reduces procurement costs compared with super-rapid recovery (SRR) with *ex-situ* NMP [51]. The estimated NRP procurement cost in the USA is $9,463.22 per donor. Conservative estimates indicate that approximately 31 donor allografts can be procured using NRP at a cost equivalent to that of a single allograft procured via SRR with *ex-situ* perfusion (Table 5) [51]. This difference stems from improved resource utilization and fewer discards (Table 5).

European economic analyses indicate that HOPE is cost-effective, with 1-year post-transplant costs lower than those of SCS, primarily due to reduced post-transplant intervention costs resulting from lower biliary complications. In a multicenter RCT in the Netherlands, the mean total cost per patient was €110,794 for HOPE versus €126,221 for SCS, with the greatest savings in intensive care unit and nonsurgical interventions [50]. Cost-effectiveness was achieved with as few as 1–30 procedures per yr, depending on whether personnel and facility costs were included, making HOPE feasible for both high- and moderate-volume centers [50] (Table 5). HOPE logistics include a perfusion device, oxygen supply, and trained staff. The protocol is straightforward and can be performed at the transplant center [50]. On January 20, 2026, the FDA granted *de novo* clearance to the Bridge to Life™ VitaSmart™ HOPE system for routine clinical use in LT in the USA [24].

### Limitations of the study

OPTN does not reliably record perfusion modality or detailed DCD agonal-phase metrics, nor does it distinguish SCS from MP. It has limited data on DCD-specific morbidity, such as biliary complications. The MP era has been shorter than the pre-MP era, potentially introducing follow-up bias into the analysis. NRP identification was based on surrogate coding and may have been subject to misclassification. The cohort was also subject to temporal misclassification bias, as era definitions based on OCS FDA approval (2021) predated the first reported NRP LTs (2022), potentially exaggerating the impact of technologies that have been broadly available the longest. Another significant limitation is the aggregation of MP modalities, despite evidence indicating that each modality has distinct mechanisms and outcomes; therefore, it remains uncertain which modality genuinely contributes to the observed improvements, aside from temporal misclassification. This latter misclassification may either amplify or obscure the impact of each modality on the outcomes, depending on the cohort’s exposure to the respective technology. Other limitations stem from the study’s retrospective design, including limited variables, residual confounding, data inaccuracies, and the inability to track changes. Additional issues include selection bias, a smaller sample size in the older group, and a higher risk of type II errors.

### Challenges and future directions

A significant challenge lies in the rising costs of organ acquisition and the uncertainty surrounding insurance coverage for these modalities [49].

Large, multicenter RCTs may be required to determine the individual benefits of different perfusion techniques in DCD LT, including older DCD donors. These studies should focus on long-term survival, quality of life, and cost-effectiveness [23, 43]. DCD LT benchmarking should be conducted prospectively, with donor–recipient risk matching, comparing risk and outcomes with the best possible results from SCS [53]. Research should also aim to standardize viability assessment criteria and perfusion protocols [23, 54, 55].

Future clinical use will likely include therapeutic interventions during MP, such as defatting steatotic livers, delivering gene therapies, or administering regenerative agents, to further enhance the quality and utilization of older DCD liver grafts [56–60]. The integration of artificial intelligence and advanced analytics to interpret perfusion data and provide decision support is also anticipated. Ongoing and future studies should also evaluate the impact of these technologies on organ allocation, waitlist mortality, and health system resource use [42, 61].

## Conclusion

MP is associated with improved utilization of older DCD liver grafts, resulting in an unprecedented increase in the use of these organs. As long-term data and access expand, MP can help recover more older DCD livers, reduce discard rates, and lower waitlist mortality. Broader adoption, guided by resources and evidence-based protocols, could bridge the gap between supply and demand in US LT and redefine acceptable donor age limits.

## Data Availability

Publicly available datasets were analyzed in this study. This data can be found here: https://unos.org/.
