# Correction: Transplantation of older DCD livers in the machine perfusion era: a U.S. cohort study

**DOI:** 10.3389/ti.2026.17169

**Published:** 2026-07-08

**Authors:** Emmanouil Giorgakis, Ioannis A. Ziogas, Dimitrios Moris, Dimitrios N. Varvoglis, Charalampos Theocharopoulos, Dor Yoeli, Megan A. Adams, Andrew S. Barbas, Martin I. Montenovo, Melissa Chen, Sorabh Kapoor, Esteban Calderon, Andrew M. Moon, Sasha Deutch-Link, Hersh Shroff, Neil D. Shah, Oren K. Fix, A. Sidney Barritt, Amit K. Mathur, Trevor L. Nydam, Chirag S. Desai, Paulo N. Martins, Andrea Schlegel

**Affiliations:** 1 Division of Abdominal Transplantation, Department of Surgery, University of North Carolina at Chapel Hill School of Medicine, Chapel Hill, NC, United States; 2 Division of Transplant Surgery, Department of Surgery, University of Colorado Anschutz Medical Campus, Children’s Hospital Colorado, Aurora, CO, United States; 3 Department of Surgery, MedStar Georgetown Transplant Institute, Washington, DC, United States; 4 Division of Abdominal Transplant Surgery, Department of Surgery, Duke University Medical Center, Durham, NC, United States; 5 Division of Hepatobiliary Surgery and Liver Transplantation, Department of Surgery, Vanderbilt University Medical Center, Nashville, TN, United States; 6 Division of Gastroenterology and Hepatology, University of North Carolina at Chapel Hill School of Medicine, Chapel Hill, NC, United States; 7 Division of Transplant Surgery, Mayo Clinic, Phoenix, Arizona, NC, United States; 8 Transplant Institute, Department of Surgery, Division of Transplantation, University of Oklahoma, Oklahoma City, OK, United States; 9 Transplant Center, Digestive Disease and Surgery Institute, Cleveland Clinic Foundation, Cleveland, OH, United States

**Keywords:** donation after circulatory death, dynamic preservation, hypothermic oxygenated perfusion, liver transplantation, machine perfusion

Author Andrea Schlegel was erroneously spelled as Andrea Schegel.

There was a mistake in the footnote of **Table 4** as published. The abbreviation for flavin mononucleotide was misspelled. The corrected footnote of **Table 4** appears below.

“COR, continuous oxygenated rewarming; DCD, donation after circulatory death; DHOPE, dual hypothermic oxygenated machine perfusion; ECMO, extracorporeal membrane oxygenation; FMN, flavin mononucleotide; HMP, hypothermic oxygenated machine perfusion; NAS, non-anastomotic strictures; NMP, normothermic machine perfusion; NRP, normothermic regional perfusion; SCS, static cold storage; PNF, primary nonfunction”.

In the abstract, errors occurred in the transfer of values from the tables to the text. This has been corrected to read:

“Normothermic regional perfusion (NRP) (3.0%–21.7%), NMP (2.3%–35.6%), and sequential NRP-NMP (0.1%–19.2%) increased significantly (p < 0.001).”

“Multivariable Cox regression showed that static cold storage (HR = 1.19), donor age 50–69 versus 18–49, and recipient age (HR = 1.04) increased the risk of graft loss after adjustment.”

Errors occurred in the transfer of values from the tables to the text.

Corrections were made to the Results section, Demographics subsection paragraphs 4 & 5:

“During our study period, total LT rates increased by 62.7% (from 8,497 LTs in 2016 to 13,824 in 2025). By the end of 2025, DCD allografts (n = 5,669) accounted for 43.2% of deceased-donor allografts (n = 13,116). In the MP era, donors ≥50 (n = 4,451) accounted for the largest share of DCD donors (61.35%) (**Figure 2**). DCD SCS dropped from 94.5% to 21.2% in the MP era, while there was a steep rise in NRP (3.0% vs. 21.7%), NMP (2.3% vs. 35.6%), and sequential NRP-NMP deployment (0.1% vs. 19.2%) (p < 0.001) (**Figure 3**, **Table 1**).

The MP-era was associated with 44.6% decrease in median waitlist time (112–62 days, p < 0.001) (**Table 1**). The primary LT indication shifted from hepatocellular carcinoma (27.2%) to alcohol-associated liver disease (35.9%) (p < 0.001). The median donor WIT increased from 23.0 to 31.0 min”

Corrections were made to the Survival outcomes subsection paragraph 3:

“In multivariable Cox regression, SCS (hazard ratio [HR] = 1.19, 95% confidence interval [95% CI]: 1.00–1.41, p = 0.045) and increasing recipient age (HR = 1.04, 95% CI: 1.03–1.04, p < 0.001) were associated with increased risk of mortality when adjusted for donor age group, WIT, donor BMI, recipient BMI, diagnosis, and laboratory MELD score (**Table 3**). SCS (HR = 1.29, 95% CI: 1.13–1.48, p < 0.001), donor age 50–69 years (vs. 18–49 years), and increasing recipient age (HR = 1.01, 95%CI: 1.01–1.02, p < 0.001) were also associated with increased risk of graft loss when adjusted for WIT, donor BMI, recipient BMI, diagnosis, and laboratory MELD score (**Table 3**).”

Corrections were made to the Discussion section paragraph 3:

“With a turning point in 2021, older DCD donors have moved from a marginal donor group to the primary driver of DCD LT volumes. In the pre-MP era, approximately 80% of DCD livers were from donors <50 years, with the remainder aged 50–69; no transplanted livers were from donors ≥70. In the MP era, the share of DCD donors aged ≥50 increased more than 2-fold (from 19.3% to 53.4%). DCD donors ≥60 rose by 7.8-fold (from 3.5% to 27.3%) (**Table 1**). The utilization of older DCD donors (≥50 years) has outpaced that of younger DCD donors, becoming the dominant DCD donor source in 2025 (n = 3,478; 61.3%) (**Figures 1, 2**).”

Authors Melissa Chen, Sorabh Kapoor, Esteban Calderon, and Chirag Desai were erroneously assigned to the affiliation Division of Transplant Surgery, Mayo Clinic, Phoenix, Arizona, NC, United States. The correct affiliation is Division of Abdominal Transplantation, Department of Surgery, University of North Carolina at Chapel Hill School of Medicine, Chapel Hill, NC, United States.

Authors Andrew M. Moon, Sasha Deutch-Link, Hersh Shroff, Neil D. Shah, Oren K. Fix, and A. Sidney Barritt were erroneously assigned to the affiliation Division of Transplant Surgery, Mayo Clinic, Phoenix, Arizona, NC, United States. The correct affiliation is Division of Gastroenterology and Hepatology, University of North Carolina at Chapel Hill School of Medicine, Chapel Hill, NC, United States.

Author Amit K Mathur was erroneously assigned the affiliation Division of Gastroenterology and Hepatology, University of North Carolina at Chapel Hill School of Medicine, Chapel Hill, NC, United States. The correct affiliation is Division of Transplant Surgery, Mayo Clinic, Phoenix, Arizona, NC, United States.

The original article has been updated.

